# Current Challenges and Future Advances in Lung Cancer: Genetics, Instrumental Diagnosis and Treatment

**DOI:** 10.3390/cancers15143710

**Published:** 2023-07-21

**Authors:** Giovanni Vicidomini

**Affiliations:** Thoracic Surgery Unit, Department of Translational Medicine, University of Campania “Luigi Vanvitelli”, 80131 Naples, Italy; giovanni.vicidomini@unicampania.it

Lung cancer is a malignancy with a poor prognosis, with only 20% of patients having an overall survival longer than five years from diagnosis, and this prognosis has still not significantly improved despite developments in understanding the genetic evolution of lung cancer; improvements in the accuracy of diagnostic procedures; and refinements in the treatments with multimodal regimens, including surgery, radiotherapy and systemic therapy (chemotherapy, immunotherapy and targeted therapy).

In this editorial, I review 10 articles presented by international leaders that focus on new potential diagnostic and therapeutic discoveries that aim to improve the prognosis of lung cancer patients. In particular, the most recent developments pertain to the biology of lung cancer, such as specific gene mutations, genomic heterogeneity and the discovery of new biomarkers.

Lung adenocarcinoma (ADC) is the most common primary lung cancer and represents about 40% of all lung cancers. Complete resection of the tumor is the best treatment, but in some subgroups of patients, particularly those with local advanced disease, a clinical response may be obtained using targeted therapies, such as tyrosine kinase inhibitors (TKIs) and immunotherapies. Unfortunately, other rare lung tumors, such as pulmonary sarcomatoid carcinoma (PSC), are extremely aggressive, poorly differentiated, and associated with poor prognosis and few treatment options. There is also a remarkable scarcity of studies on human patients, which makes the development of preclinical models of crucial importance for understanding the disease and for developing novel therapeutic approaches. The article by Lázaro et al. [1] reported the first mouse model of PSC available for preclinical research. In particular, the authors evaluated the role of the combined deletion of the tumor suppressors Pten and Trp53 in adult mice lungs. This work showed that the combined deletion of Pten and Trp53 leads to the development of ADC and PSC, irrespective of the targeted cell type in which the gene alterations initially occur, at least after naphthalene treatment. This experimental model sheds light on the relationships between ADC and PSC, and their cells of origin. Moreover, human ADC shows strong transcriptomic similarities to the mouse PSC, providing a link between both tumor types and the human ADC. Unfortunately, despite numerous similar reports in the literature, the biological characteristics of PSC remain generally unclear. For this reason, the treatment of this and other rare lung cancers still faces huge challenges. Therefore, researchers in this field need to collectively make efforts to detect more molecular alterations and to explore new promising treatments that may bring maximal benefit to patients.

In another paper, Otake et al. [2] focused their research on the diagnostic role of cell-free DNA released from lung cancer cells in the airway. Although bronchoscopy is considered a mandatory procedure for diagnosing lung cancer, its diagnostic yield remains unsatisfactory and is reported to be approximately between 40%-60% for cancers located in the peripheral lungs. Although innovative technologies, including ultrathin fiberscope, navigation systems, endobronchial ultrasound and new testing systems (ROSE: rapid onsite cytology) have improved the diagnostic yield in recent years, further improvement is necessary. Assuming that lung cancer cells release cell-free DNA into the epithelial lining fluid, the authors hypothesized that lung cancer could be diagnosed by analyzing gene mutations in cell-free DNA collected by means of bronchoscopic bronchoalveolar lavage (BAL), a method used to diagnose interstitial pneumonia and respiratory infection in general respiratory practice. However, BAL is not generally performed for diagnosing lung cancer because it has been occasionally reported to be associated with different complications, such as respiratory failure, pneumonia and bronchial asthma. For this reason, they developed an ex vivo BAL model (bronchoalveolar lavage performed on the resected lung samples) after lobectomy to explore and evaluate the possibility of lung cancer diagnosis using BAL fluid samples and to simulate the actual clinical practice of BAL. This study included 32 patients with lung cancer who underwent surgery. Each DNA sample (i.e., BAL fluid, primary lesion and plasma) underwent deep targeted sequencing. Gene mutation analyses in the BAL fluid samples identified mutations identical to those in the primary lesions in 30 of 32 patients (93.8%). In contrast, the microscopic cytology of the same BAL fluid samples yielded a diagnosis of lung cancer in only one of 32 patients, and the analysis of plasma samples revealed gene mutations identical to those in the primary lesions in only one of 32 patients. Based on these results, the authors concluded that cell-free DNA released from lung cancer cells exists more abundantly in the airway than in the blood. The collection and analysis of the BAL fluid containing cell-free DNA derived from lung cancer can thus allow for lung cancer diagnosis and the screening of driver mutations. Although the physiological mechanisms and efficiency of DNA release from tumors into the airway remain to be clearly elucidated, I believe that this method, if validated by other studies, could have several diagnostic positive implications, simplifying the detection of lung cancer and the monitoring of treatment efficacy and genetic tumor changes over time.

About current therapies used to treat lung cancer, only one quarter of patients with NSCLC are diagnosed with an early stage of the disease and judged to be eligible for curative-intent surgery. Nevertheless, prognosis remains disappointing for those who regularly experience relapses. With a five-year median follow-up, the percentage of people who have disease recurrence or die after surgery ranges from 45% among patients with stage IB disease to 76% among those with stage III disease, regardless of the use of postoperative systemic therapy. Patients diagnosed with advanced NSCLC are systemically treated with chemotherapy, immunotherapy and targeted therapy, depending on the genetic background of the disease. Among the targeted treatments in patients with advanced NSCLC excluded from surgery, the tyrosine kinase inhibitor (TKI) osimertinib is the standard of care for non-small cell lung cancer (NSCLC) patients with activating mutations in the epidermal growth factor receptor (EGFR). However, osimertinib has an elevated risk of developing resistance to the treatment. A substantial fraction of the mechanisms for resistance is unknown and may involve RNA and/or protein alterations. In their study, Kosibaty et al. [3] investigated the full transcriptome of parental and osimertinib-resistant cell lines, revealing 131 differentially expressed genes. Knockdown screening of the genes upregulated in resistant cell lines uncovered eight genes that partly confer resistance to osimertinib. Among them, they detected the expression of Ras-related protein Rab-32 (RAB32) and thrombospondin 1 (THBS1) in plasmas sampled at baseline and at disease progression from EGFR-positive NSCLC patients treated with osimertinib. Both genes were upregulated in the progression samples. Moreover, they found that the knockdown of RAB32 and THBS1 reduced the expression of phosphorylated focal adhesion kinase (FAK). A combination of osimertinib with a FAK inhibitor resulted in synergistic toxicity in osimertinib-resistant cells, suggesting a potential therapeutic drug combination for overcoming resistance to osimertinib in NSCLC patients. These results indicate a need to identify biomarker-matched treatments to target specific mechanisms of resistance or to prevent the emergence of these mechanisms. Several ongoing studies aim to provide evidence for the potential use of osimertinib in combination with other drugs, with promising preliminary results. Moreover, in patients with non-small-cell lung cancer (NSCLC) harboring epidermal growth factor receptor (EGFR) mutations, brain metastasis is a factor influencing poor prognosis. The standard treatment is systemic therapy combined with intracranial intervention, such as craniotomy or radiotherapy. However, intracranial intervention may result in neurological or cognitive deficiency. In addition, NSCLC patients with brain metastasis may show poor responsiveness to chemotherapy. In this regard, Kuo et al. [4] conducted a retrospective study to determine the optimal treatment strategy for EGFR-mutant NSCLC patients with brain metastasis receiving or not receiving intracranial intervention. Intracranial intervention had no statistically significant impact on response rate (RR), progression-free survival (PFS) or overall survival (OS) of patients with EGFR mutations and brain metastasis who received EGFR tyrosine kinase inhibitors (TKIs) as a first-line therapy. Treatment with different EGFR TKIs did not result in significant differences in RR or OS, but PFS differed significantly between the therapies. Among the TKIs used, afatinib and osimertinib both demonstrated significantly longer PFS than gefitinib. In conclusion, the authors suggest that intracranial intervention is not necessary in this subgroup of patients. This study showed that the management of brain metastases in lung cancer is still a challenge and that systemic therapies have a central role in this subgroup of patients. To fully optimize the tools currently available for treating intracranial metastases, further prospective studies are needed to evaluate the most effective combinations of novel systemic therapies. The goal for intracranial management should be to better understand how tumor mutations and other tumor-specific factors might inform clinical decision-making in the modern treatment era.

In the recent decade, another systemic therapy, immunotherapy by checkpoint inhibitors (ICIs), has reshaped the treatment of advanced lung cancer. Three immune checkpoint pathways, namely programmed cell death-1 (PD-1), programmed cell death-ligand 1 (PD-L1) and cytotoxic T-lymphocyte antigen 4 CTLA-4, have emerged targets for cancer therapeutics. In patients with non-oncogene addicted advanced NSCLC, the PD-1/PD-L1 blockade is currently the standard of care as a monotherapy or in combination with chemotherapy, according to PD-L1 expression level. Immunotherapy with anti-PD-L1 durvalumab after curative chemoradiation has demonstrated prolonged survival in patients with stage III unresectable NSCLC. However, PD-L1 tumor tissue expression is a limited predictor of anti-PD-1 efficacy. In some clinical trials, it has been demonstrated that the greater the expression of PD-L1, the greater the observed response. However, in other studies, this association was not observed. In their study, Rogado et al. [5] prospectively explored baseline peripheral blood mononuclear cells (PBMCs) to assess immunotherapy predictors. They included 39 patients diagnosed with non-small cell lung cancer and treated with immunotherapy in the study group and 40 patients with advanced malignancies treated with a non-immunotherapy treatment, as the control group. In these patients, they detected that high baseline levels of circulating T cell subpopulations (in particular, CCR9, CCR10 and CXCR4) related to tissue lymphocyte recruitment are associated with poorer outcomes for immunotherapy-treated advanced non-small cell lung cancer patients, and these differences were specific to immunotherapy-treated patients. This study shows that the road to identifying immunotherapy predictors that can predict the validity of immunotherapy in lung cancer is still long. However, reports in the literature indicate that research on chemokine receptors is crucial for a better understanding of the mechanisms underlying the efficacy of this promising therapy.

Another study by Grenda et al. [6] evaluated why some patients do not benefit from immunotherapy despite high PD-L1 expression on their tumor cells, while others respond to immunotherapy despite a lack of expression of this molecule. Data from the literature indicate that various bacteria may favor the effectiveness of immunotherapy or may be involved in its failure. The authors paid attention to the composition of the gut microbiome as a potential predictive factor for immunotherapy effectiveness. They analyzed the intestinal microbiome composition of 47 patients with stage IIIB or IV NSCLC and assessed how this composition affects the efficacy of ICI treatment as monotherapy or in combination with chemotherapy. The results show that the composition of the intestinal microbiome is important in the estimation of the immunotherapy effectiveness and the occurrence of ICI toxicity in patients with advanced non-small cell lung cancer. They found that a high abundance of Bacteroidaaceae, Barnesiellaceae and Tannerellaceae could extend PFS; moreover, the risk of death was significantly higher in patients with high amounts of bacteria from the Ruminococcaceae family and in patients with a low abundance of Clostridia. However, the results regarding microbiome composition and response to immunotherapy can be inconclusive due to differences in the type of cancer, bacterial synergism, patient diet or ethnicity. The study suggests how numerous and unclear the factors affecting the efficacy of immunotherapy are. This indicates a strong need for more in-depth research into different areas.

However, with the success of immune checkpoint inhibitors in metastatic lung cancer, immunotherapy has shown great potential in the resectable early stages in making an often lethal disease, due to its frequent post-surgery relapses, more curable. Following the implementation of ICIs in the management of locally advanced and metastatic NSCLC, several studies on neoadjuvant or adjuvant immunotherapy have been carried out in recent years. In this regard, a review by Viscardi et al. [7] focused on the change in the treatment paradigm of early-stage NSCLC due to the advent of ICIs. They evaluated the potential role of two recently approved immune checkpoint inhibitors for early-stage NSCLC: anti-PD-L1 atezolizumab for the adjuvant treatment of stage II to IIIA resected NSCLC and anti-PD-1 nivolumab in combination with platinum-based chemotherapy for the preoperative treatment of resectable NSCLC. The authors concluded that potential the advantages of preoperative immunotherapy with nivolumab include better compliance and assessments of treatment efficacy prior to surgery, as well as the treatment of micro-metastases as early as possible. However, areas of uncertainty remain, including the optimal duration of therapy, the criteria for de-escalation or escalation after neoadjuvant immunotherapy and surgery, and the roles of chemotherapy and radiation. Moreover, the results of this study and other data from the literature suggest that a preoperative approach would be preferable in high-risk patients or for more advanced stages, whereas adjuvant therapy could be an alternative when delaying surgery is not advised. The complexity of the therapeutic decisions to be made demonstrates how valuable a precocious molecular profiling is in early-stage NSCLC and even more so at the borderline stage IIIA-N2, which is the stage at which the disease is determined to be potentially resectable or not, with the present definition of resectability being largely dependent on local expertise. This also indicates the importance of evaluating these patients in a multidisciplinary meeting to define the most valuable treatment strategy.

Regarding the prognosis of lung cancer patients undergoing surgery, several studies have demonstrated a potential interconnection between inflammation and poor prognosis of cancer. In broader terms, inflammatory status could impact the quality of life of the patients in terms of their immune response to cancer, particularly on the metabolism of lung cancer and of the host. In their study, Mazzella et al. [8] investigated if and how pre-operative inflammatory status can influence the long-term prognosis of patients undergoing lung surgery for cancer. The inflammatory pre-operative statuses of 257 patients were investigated by calculating their albumin levels; their CPR (c-protein reactive) levels; their complete blood counts; and some other indexes related to inflammatory status, namely the HALP amalgamated index, the platelet-to-lymphocyte ratio (PLR), the neutrophil-to-lymphocyte ratio (NLR), the systemic immune-inflammation index (SII) and the advanced lung cancer inflammation index (ALI). The authors found a strong association between the different analyzed inflammatory indexes and poor prognosis. In addition, they analyzed and found, for the first time, correlations between some other parameters—such as ALI, SII and HALP—and the long-term prognosis of resected lung cancer, and concluded that pre-operative inflammatory status strongly influences long-term prognosis in patients affected by NSCLC and undergoing surgery. For this reason, research should focus on the evaluation of possible therapeutic measures capable of modifying inflammatory status and thus improving the prognosis of these patients.

Moreover, the consuming character of lung cancer and the debilitating multimodal therapeutic regimens used to improve patients’ prognoses often cause high symptom burden and loss in functional capacity and quality of life in the patients. In addition, lung cancer patients frequently have cardiac or pulmonary comorbidities that lead to reduced exercise performance and physical activity. Pulmonary rehabilitation is a multidisciplinary intervention that aims to reduce functional impairment, symptoms and disability in people with lung disease, especially chronic obstructive pulmonary disease (COPD). The rehabilitation process consists of physical training, disease education, and nutritional and psychological counseling, along with social and behavioral interventions. It is still under debate if, when and in which form a pulmonary rehabilitation program may improve exercise performance and reduce symptom load in lung cancer patients after surgery. Illini et al. [9] have performed a retrospective analysis of surgically resected lung cancer patients who underwent a 6-week multi-professional outpatient pulmonary rehabilitation (OPR) program. Fifty-seven patients were included in the study. At completion of OPR, there was a statistically significant mean increase of 50 m in six MWTs. Significant improvements were also seen in all other exercise and strength tests, accompanied by a significant reduction in the COPD Assessment Test (CAT) score, which was used to assess COPD symptom burden. The authors concluded that OPR seems to be an effective and safe intervention in surgically treated lung cancer patients that can improve overall quality of life and outcomes. The results of this study justify the increasing attention of research towards preoperative measures that improve the preoperative physical state of patients with lung cancer and, thus, increase the treatment outcomes, especially those of surgery.

As lung cancer represents the most common primary tumor associated with central nervous system (CNS) metastases, accounting for up to 50% of the cases, D’Aiello et al. [10] provided an overview on recent advances in the management of brain metastases including novel radiotherapy techniques, targeted agents with demonstrated CNS activity, evidence for immune checkpoint inhibitors and strategies for treating leptomeningeal disease. Stereotactic radiology surgery (SRS) has emerged as an effective radiotherapy technique with fewer toxicities compared with whole brain radiotherapy (WBRT). Furthermore, multi-generation tyrosine kinase inhibitors (TKIs) with overall CNS response rates (ORR) of up to 70–80% are now an accepted first-line approach for a subset of advanced NSCLC patients with targetable molecular alterations. In addition, while the CNS was once considered an immunologic sanctuary site, growing evidence shows that immune checkpoint inhibitors (ICIs) can induce durable responses in brain metastases as well. Given the complex nature of managing CNS metastases in NSCLC, often requiring multidisciplinary management, the authors propose a simplified decision tree that may be of use for management of this subgroup of patients. However, they conclude that continued progress is required to standardize treatment approaches in NSCLC patients with CNS metastases. But, what are the possible directions for future research? Progress in the treatment of NSCLC metastatic to the brain will be dependent on a better understanding of the biological basis of CNS metastases. Moreover, we need improved trial designs with deliberate inclusion of CNS-specific outcomes. Currently, various preclinical studies have investigated how novel agents might be applied to minimize metastasis. Other ongoing areas of investigation aim to identify molecular alterations that may be specifically associated with CNS metastases and that are potentially targetable. Thus, further efforts are necessary to identify other targets and treatments for NSCLC CNS metastases.

In conclusion, this Special Issue of *Cancers* is a collection of very interesting articles discussing the role of new diagnostic and therapeutic developments in lung cancer, particularly those regarding the biology of lung cancer, targeted therapy, immunotherapy and other achievements that could improve the prognosis of patients. However, more work is needed to increase our knowledge about all aspects of this threatening neoplastic disease.
